# Mixed Reality for Cross-Cultural Integration: Using Positive Technology to Share Experiences and Promote Communication

**DOI:** 10.3389/fpsyg.2018.01223

**Published:** 2018-07-17

**Authors:** Annamaria Recupero, Stefano Triberti, Camilla Modesti, Alessandra Talamo

**Affiliations:** ^1^Department of Social and Developmental Psychology, Sapienza University of Rome, Rome, Italy; ^2^Department of Psychology, Università Cattolica del Sacro Cuore, Milan, Italy; ^3^Department of Oncology and Hemato-Oncology, University of Milan, Milan, Italy

**Keywords:** immigration, mixed reality, augmented reality, positive technology, integration

In the contemporary society, migrations from other countries or even from other regions of the country are a common phenomenon that rises cultural and psycho-social issues, as well as political and economic challenges. People move from their place of origin for educational or professional purposes or because they are forced to leave due to political, economic and social conditions, and also natural disasters which produce population flows. Whatever the push and pull factors are, when people move permanently or temporarily they tend to maintain close ties with their place of origin (with people, places, culture, practices etc.), while trying to develop attachment with the place of residence (Ehrkamp, 2005).

The literature (Appadurai, 1996; Faist, 2000; Smith, 2001) highlighted the dichotomy between transnational and local levels, arguing that transnational ties may prevent assimilation and adaptation to the new place, while other authors (Bhabha, 1994; Kaya, 2002; Ehrkamp, 2005) promoted the concept of “hybridity” between home and host society, or between ethnicity and assimilation, showing that immigrants' transnational practices create new places of belonging that allow them to engage with the receiving society.

According to Berry (2001), four strategies are mainly used by immigrants interacting with the culture of the hosting country:

- **Assimilation**, where the person prefers not to maintain his cultural heritage and seek continuous interactions with the culture of the hosting country;
- **Separation**, where the person tries to preserve the attachment to the culture of origin and avoid the contact with the culture of the hosting country;
- **Integration**, where the person tries to engage within both cultures;
- **Marginalization**, or detachment from both cultures

As Ehrkamp says, “Conceptualizing migrants' identities as constantly negotiated in relation to multiple societies and places enables us to think beyond dichotomies and mutually exclusive notions of local and transnational ties, and to recognize immigrants as agents who are able to forge their belonging and multiple attachments” (Ehrkamp, 2005, p. 348). Indeed, people have a cultural identity or a “set of beliefs and attitudes themselves in relation to culture group membership” (Berry, 2001 p. 620); usually one becomes aware of his/her own cultural identity when coming in contact with people from other cultures (Phinney, 1990; Berry, 1999). In other words, immigrants construct their identities in the context of a negotiation between old and new homes' contexts.

However, such a process is not free from obstacles and issues, with notable consequences on immigrants' well-being. According to literature, some psychosocial issues can be identified regarding identity re-negotiation while moving to a different place, and cultural integration.

First of all, immigrants could experience feeling of isolation, estrangement and alienation, related to the difficulty to create strong social ties in the new place (Hurtado-de-Mendoza et al., 2014), with consequences ranging from negative emotions to depression and other serious treats to health (Lackey, 2008); such risks can be reduced by being part of the community, belonging, and contributing to the community development (Esser, 2001).

Other important aspects are the strains and stresses associated to practical issues such as adapting to new culture, language, ways of doing things, and/or dealing with different practices and new institutions (Takyi, 2002; Perreira et al., 2006), a process sometimes labeled “acculturation stress” (Birman et al., 2007).

Moreover, homesickness is a frequent phenomenon associated with leaving one's home, either temporarily or permanently, voluntarily or in some forced manner. Several studies (for a recent review see: Stroebe et al., 2015) suggest that homesick individuals can experience substantial distress and are at increased risk for psychological and physical health problems and lowered well-being. Indeed, homesickness correlates range from emotional (e.g., yearning, loneliness), cognitive (e.g., exaggerate concerns and intrusive toughts about home and attachment figures), social (e.g., withdrawing from relationships in the new environment), to somatic reactions (e.g., loss of weight and appetite). In the context of immigrants' experience, and especially that of refugees, traumatizing experiences may establish, with negative consequences ranging from emotional suffering to psychological disorders, but also positive ones such as the development of new resources (i.e., adversity activated development) (Papadopoulos, 2007).

Finally, it should be noticed that integration is not desirable for emotional well-being only: immigrants need to integrate in the hosting country for practical reasons too, for example getting education (Zhou and Kim, 2006), or participating in shared decision making regarding healthcare (Cutica et al., 2014; Renzi et al., 2016).

The purpose of the present opinion is to highlight some innovative resources to deal with these challenges, starting from the positive technology paradigm and, more specifically, from the concept of mixed reality. Broadly speaking, Positive technology is a theoretical and applied approach that considers human health and well-being as the main objective for technological advancement (Riva et al., 2012). It is not a mere philosophical stance; on the contrary, positive technology offers guidelines for designing technology, in order to pursue specific well-being outcomes. In a broad sense, technology may be used to structure, augment or replace user experience with digital content; also, positive devices may be used to promote positive emotions (hedonic technology), to support the user in the achievement of engaging and self-actualizing experiences (eudaimonic technology), and to enhance connectedness among individuals, groups and societies (social-interpersonal technologies).

Since the first theorization of the positive technology paradigm, digital and immersive devices have been considered some of the most advanced and promising tools for promoting health and well-being (Riva et al., 2016). Specifically, virtual reality and augmented reality are able to (1) expose users to stimuli and complex situations that are normally impossible to reproduce in a physical laboratory or clinical setting; (2) are characterized by inherently engaging properties (e.g., users often perceive them as interesting, funny, intriguing, and may prefer them over more traditional devices); (3) are scalable and adaptable to different contexts and issues, in that virtual environments and digital stimuli can be designed *ad-hoc*.

The term “mixed reality” has been used to identify situations in which digital/synthetic elements co-exist with real ones (Milgram and Kishino, 1994), “somewhere in the middle” between only-physical and totally-immersive virtual environments. Although the positive technology paradigm refuses any extreme idea of the so-called “virtual reality continuum” (because virtual experiences could be “real” from a subjective viewpoint no less than digital ones, and they could have “real” positive and negative effects on users' health and behavior), this term is still used as a broad label including any user experience in which physical or digital elements are not predominant in an absolute sense, but rather an admixture of the two can be envisaged. This points to augmented reality (AR), or the superimposition of digital objects on physical environments and/or composition of the two, which users perceive thanks to equipped devices ranging from the smartphone to dedicated digital glasses.

What has AR to do with the cross-cultural integration issue? Augmented Reality is widely used in fields ranging from medical, military and entertainment (Azuma, 1997; Berryman, 2012), while in socio-psychological interventions its usage is still generally limited to exposure therapy (Chicchi Giglioli et al., 2015).

It can be said that some of the main issues in immigrants' experience, which can generate externalized (e.g., prejudice, unfair social treatment) and internalized problems (e.g., depression, isolation, negative emotions) can be described in terms of lack of something in the new environments. First of all, there is the lack of people, places, practices, and objects from home; this can be contextualized in terms of homesickness and refusal of the new place. Secondly, there may be lack of skills and/or opportunities to communicate with new people as well as with new institutions, which are fundamental factors in the integration process.

In a general sense, AR could provide resources for intervention in that it is based on the *addition* of digital elements to the physical environment, instead of its substitution with an immersive experience which, in this case, may act as a palliative care for sadness but does not help to integrate oneself in a new, “real” physical environment and social context. This relates to the fact that social and mobile technology became “a cornerstone of immigration experience,” with immigrant families making an extremely frequent use of computer-mediated communication with their distant relatives about daily decisions and life cycle core issues (Bacigalupe and Cámara, 2012). Indeed, people use any possible means for communication when they feel a urgent need for contact and social support (Wellman et al., 2001), and the use of social media is common among immigrants to maintain contact both with people and community identity (Mallapragada, 2013), to the point that media usage seem not to dissolve geographical identity or connectivity, but rather it reinforces them (Van Den Bos and Nell, 2006).

On the basis of such a widespread usage of technologies among immigrants, new technologies can be used to provide new resources both for dealing with distress and promoting integration.

AR-based Positive technologies can help to maintain the relation with the home country, and also to foster the inclusion in and attachment to the receiving society, by providing users with sources of identification that stretch beyond the national and local contexts of their old and new homes. Addressing social connectedness, mixed reality can provide the medium to share the meanings people attach to places, people and cultures, and creating belonging in the receiving society. Indeed, people can better approach the receiving society by understanding the cultural meanings connected with places, history, and activities.

An example can be derived from the NostalgiaBits project (Morganti et al., 2013), that aimed to enhance the intergenerational relationships (i.e., elderly and youngsters) through the sharing of experiences and memories about places and circumstances. Based on an integrated web-based platform, this project effectively improved young users' representation of the elderly as well as the elderly's well-being by making available new affective and social outcomes (Morganti et al., 2016). Indeed, technologies based on sharing memories/experiences can be used to improve both social and emotional well-being (Giorgi et al., 2013; Talamo et al., 2017).

Such concept can be further explored using AR, by providing the digital medium to enable exchanges between people of different ages, or with different cultural backgrounds.

Specifically, users could share reminiscences, experiences, or even future projects by superimposing personalized images (e.g., pictures, drawings…) on environmental features they are observing together, on site or at a distance. Such a sharing technology, based on the augmented visualization of past or future objects in the environment, may be used for example to combat homesickness (Scopelliti and Tiberio, 2010), or to favor integration among different cultural backgrounds, in that experience sharing is proven to promote empathy and perspective taking and to reduce prejudice (Pettigrew and Tropp, 2008).

Figure 1 resumes the ideas highlighted here as resources to address immigrants' emotional and social issues.

**Figure 1:**
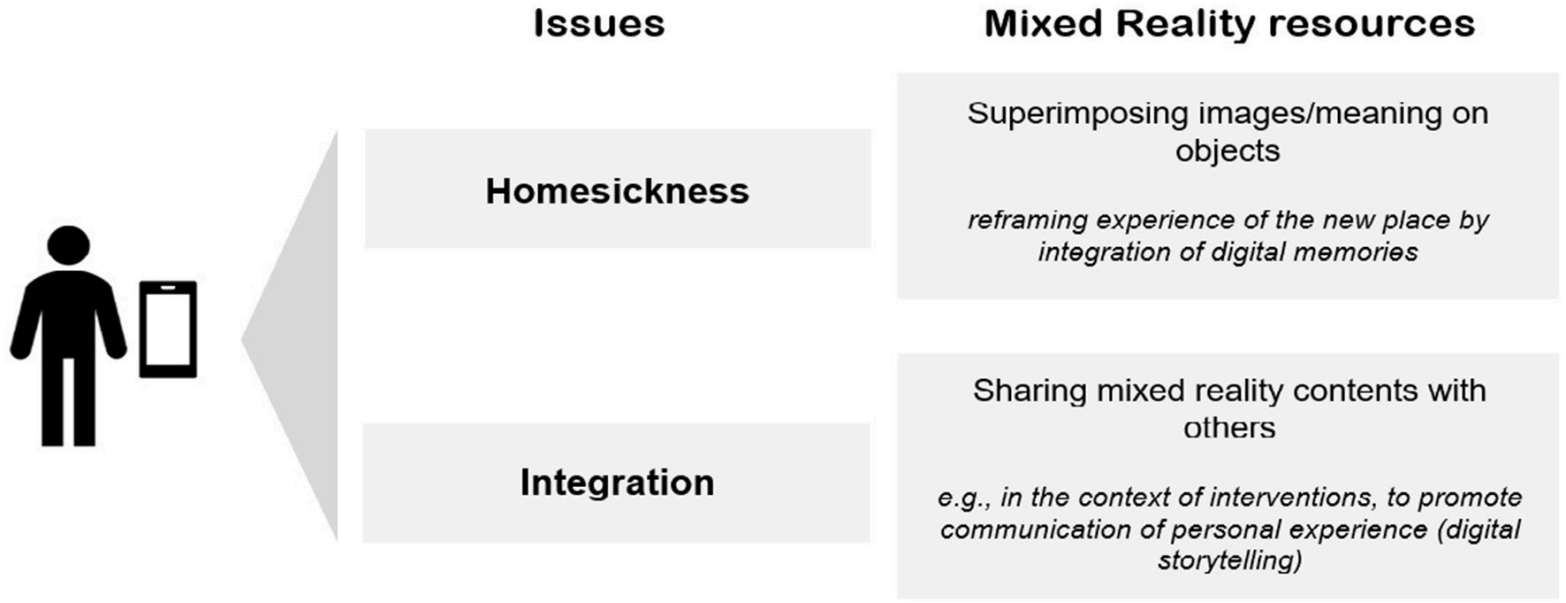
Resume of mixed reality resources for addressing integration issues.

The process of social inclusion can be fostered by enabling meaningful practices that include the social, cultural, and emotional aspects in immigrants' intercultural communication with people in the receiving culture.

To sum up, the proposed concept:

- acts on how the new environment is perceived, by “revealing” the cultural meanings, the practices, memories and personal representations developed by the community;
- builds virtual bridges, to decrease immigrants' sense of distance from their countries of origin and feelings of isolation;
- is based on digital storytelling as a practice to make meaning and share experiences of places, events and people (Alexandra, 2008).

In storytelling, by the process of re/considering and actively re-constituting stories, a sense of agency is constructed against disempowering circumstances (Jackson, 2002). Immigrants use narratives and share memories of the homeland to re-affirm their identities (Ramsden and Ridge, 2013; Lenette et al., 2015). Reminiscence and storytelling involving the communities and neighborhoods promote exchanges, mutual understanding, and respect between different age- and cultural-groups (Mercken, 2002).

The proposal expressed in the present opinion article is still in its infancy. However, it provides an innovative idea for positive technology (social-interpersonal), which may guide the development of future devices and applications to enhance health and well-being in the growing population looking for a new life in places distant from home.

This idea deserves future research, not only for technical development, but also regarding its inclusion into psycho-social interventions for integration; these would be focused on structuring and regulating its usage. Indeed, mixed reality-based shared storytelling may feature some shortcomings; for example, it may be used among immigrants only, this way reinforcing intragroup processes that promote nostalgic complacency, and prevent immigrants to build new resources for integration in the hosting culture and social context. Social-interpersonal positive technologies should be managed from design to intervention, in order to make use of their affordances for social inclusion of new relationships with outgroup members.

## Author contributions

AR conceptualized the work. AR and ST drafted the paper. CM contributed with discussion about issues related to migration and immigrants' experience. AT revised the paper and supervised the whole process. All the authors provide approval for publication of the content.

### Conflict of interest statement

The authors declare that the research was conducted in the absence of any commercial or financial relationships that could be construed as a potential conflict of interest.
